# Host genetics exerts lifelong effects upon hindgut microbiota and its association with bovine growth and immunity

**DOI:** 10.1038/s41396-021-00925-x

**Published:** 2021-03-01

**Authors:** Peixin Fan, Corwin D. Nelson, J. Danny Driver, Mauricio A. Elzo, Francisco Peñagaricano, Kwangcheol C. Jeong

**Affiliations:** 1 Emerging Pathogens Institute, University of Florida, Gainesville, FL USA; 2 Department of Animal Sciences, University of Florida, Gainesville, FL USA; 3 Department of Animal and Dairy Sciences, University of Wisconsin-Madison, Madison, WI USA

**Keywords:** Microbiome, Agricultural genetics

## Abstract

The gut microbiota is a complex ecological community that plays multiple critical roles within a host. Known intrinsic and extrinsic factors affect gut microbiota structure, but the influence of host genetics is understudied. To investigate the role of host genetics upon the gut microbiota structure, we performed a longitudinal study in which we evaluated the hindgut microbiota and its association with animal growth and immunity across life. We evaluated three different growth stages in an Angus-Brahman multibreed population with a graduated spectrum of genetic variation, raised under variable environmental conditions and diets. We found the gut microbiota structure was changed significantly during growth when preweaning, and fattening calves experienced large variations in diet and environmental changes. However, regardless of the growth stage, we found gut microbiota is significantly influenced by breed composition throughout life. Host genetics explained the relative abundances of 52.2%, 40.0%, and 37.3% of core bacterial taxa at the genus level in preweaning, postweaning, and fattening calves, respectively. *Sutterella, Oscillospira*, and *Roseburia* were consistently associated with breed composition at these three growth stages. Especially, butyrate-producing bacteria, *Roseburia* and *Oscillospira*, were associated with nine single-nucleotide polymorphisms (SNPs) located in genes involved in the regulation of host immunity and metabolism in the hindgut. Furthermore, minor allele frequency analysis found breed-associated SNPs in the short-chain fatty acids (SCFAs) receptor genes that promote anti-inflammation and enhance intestinal epithelial barrier functions. Our findings provide evidence of dynamic and lifelong host genetic effects upon gut microbiota, regardless of growth stages. We propose that diet, environmental changes, and genetic components may explain observed variation in critical hindgut microbiota throughout life.

## Introduction

The gut microbial community is dynamic and complex. It is populated by trillions of microorganisms that consist of >1000 bacterial species. This system of organisms coevolved with hosts and provides fundamental functions such as regulating host metabolic and immune pathways and preventing pathogen colonization [1, 2]. Variation within the gut microbiota is driven by intrinsic factors, such as host genetics [3], age [4], and sex [5] as well as extrinsic factors, such as diet [6], lifestyle [7], and environmental conditions [8]. Prior research establishes that diet and environmental factors have dominant roles in shaping the gut microbiota [8, 9].

Although a growing number of studies across animal species have demonstrated the determining role of host genetics upon the composition of gut microbiota, our understanding regarding the potential effect of the host genetics upon the gut microbiota composition and its sequential influences on host physiology remains limited. This limitation is due to the difficulty with controlling population variation, genetic distance, age, and environmental conditions, as well as confounding effects between these factors. Recent advances in genomics, especially genome-wide association studies (GWAS), are helping to provide mounting evidence that host genetics plays a critical role in shaping the gut microbiota. For example, Zhang et al. [10] recently found that host genetics accounts for approximately 0–41% of variation on the abundance of microbiota composition in the rumen of dairy cows; and they also identified microbiota associated with host genes *DNAH9*, *ABS4*, and *DNAJC10*. Further, Li et al. [11] found heritable rumen microorganisms that were associated with host feed efficiency and volatile fatty acid measures, as well as 19 single-nucleotide polymorphisms (SNPs) associated with rumen microbial taxa in beef cattle.

We recently evaluated host genetic effects upon the early stage of hindgut microbiota development in 3 months old juvenile cattle with a graduated spectrum of breed compositions that ranged from 100% Angus to 100% Brahman [12]. The multibreed Angus-Brahman (MAB) population was bred and raised under the same environmental conditions and fed identical diets. Thus, individual variation in the early stage gut microbiota was primarily explained by genetic differences within the hosts [12]. Interestingly, numerous butyrate-producing bacteria were associated with breed composition and potentially contributed to the weight gain of juvenile cattle [12]. Mucin-degrading bacteria competing with butyrate-producing bacteria in the GI tract were strongly associated with four SNPs located in mucin-encoding genes (*MUC4*, *MUC12*, *MUC13*, and *MUC20*); these findings support the theory that host genetics affects the gut microbiota in juvenile cattle in which the rumen is not fully developed [12].

The effect of host genetics on the gut microbiota is a promising area of research, which has opened new avenues to potentially cure certain human genetic diseases by targeting heritable bacteria. For example, the genetic risk score for inflammatory bowel disease is associated with a decrease in *Roseburia* [13], a bacteria that can promote regulatory T-cell differentiation and maintain tight junction integrity in colitis [14, 15]. Further, animal breeding and genetic selection can target animals with a desired microbiota composition to foster greater feed efficiency or to lower methane emissions [10, 16]. However, recent findings challenge the statistical significance of associations between host SNPs and bacterial taxa in a chicken model, and also show that influences of the environment and diet dominate host genetics in shaping the gut microbiota [8, 12, 17]. This uncertainty is due in part to the difficulty of controlling extrinsic factors that influence the microbial community among individuals, such as variations in diet, age, and environmental conditions. Therefore, the degree to which host genetics affects the gut microbiota, and its roles on host physiological traits, has not reached consensus [8, 9, 18, 19].

In this study, we hypothesized that repeated measures of the gut microbiota in a cohort of animals with different genetic backgrounds raised under variable diet and environmental conditions would reveal readily apparent host genetic effects on gut microbiota development. To test this hypothesis, we deepened the understanding of host genetic effects in shaping the hindgut microbiota using the MAB population throughout the production lifecycle. We used a cohort of multibreed cattle with a graduated spectrum of Angus and Brahman composition. We systematically evaluated the gut microbiota at three different growth stages: preweaning, postweaning, and fattening stages to understand host genetic effects in relation to diet and environmental changes on the hindgut microbiota. We also investigated how host genetic effects upon gut microbiota may affect animal growth rates and immune responses by identifying specific host genes at different growth stages that are known to be associated with bacteria related to weight gain and immunity.

## Materials and methods

### Ethics statement

All animal operations in this study followed the standard practices of animal care and use. Practices related to the animals in this study were approved by the University of Florida Institutional Animal Care and Use Committee (IACUC number 201408629 & 201803744).

### Animal genetic background and management

Animals used in this study were from one generation of the multibreed Angus-Brahman (MAB) beef cattle population at the University of Florida as described previously [12]. Cattle were assigned to six breed groups (BGs) according to breed composition estimated from a documented pedigree: BG1 (100 to 80% Angus and 0 to 20% Brahman), BG2 (79 to 60% Angus and 21 to 40% Brahman), BG3 (62.5% Angus and 37.5% Brahman), BG4 (59 to 40% Angus and 41 to 60% Brahman), BG5 (39 to 20% Angus and 61 to 80% Brahman), and BG6 (19 to 0% Angus, and 81 to 100% Brahman). Mating in the MAB population followed a diallel design where sires from each of the six BGs were mated to dams from all six BGs.

**Fig. 1:**
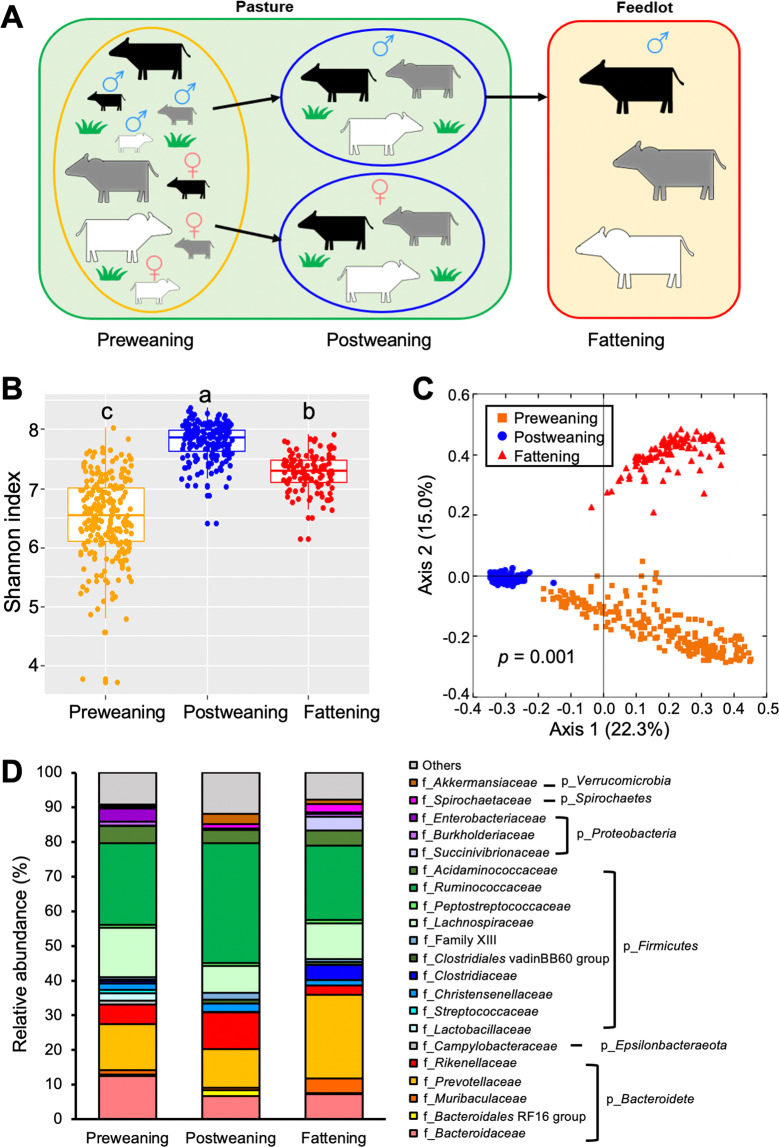
Dynamics of gut microbiota in a multibreed Angus-Brahman cattle population throughout life. **A** Animal management in the multibreed Angus-Brahman population throughout life. After birth, preweaning calves (*n* = 239) were raised on pasture together with their mothers. Postweaning steers (*n* = 100) and heifers (*n* = 95) were raised on pasture in separated pens. Subsequently, 1-year-old steers (*n* = 105) were moved to a contracted feedlot. **B** Shannon index across different stages. Different superscripts denote significant differences (*p* < 0.05), based on the one-way ANOVA followed by Tukey’s HSD test for pairwise comparison of multiple means. **C** PCoA plot based on Bray–Curtis distances comparing gut microbiota composition across the three different growth stages. Differences in Bray–Curtis distance among growth stages were analyzed using PERMANOVA. **D** Distribution of abundant bacterial families (relative abundance >1%) across different growth stages.

A total of 278 MAB calves were naturally born on pasture during the calving season. Environmental conditions including farm management and diets across BGs were identical at each growth stage. During the preweaning stage, calves were kept at the Beef Research Unit (BRU) in Waldo, FL, and were raised with their dams on the same bahiagrass (*Paspalum notatum*) pastures. Calves were weaned at ~9 months of age. Heifers and steers were placed in two separate bahiagrass pastures after weaning at the BRU. Postweaning steers were supplied with a higher level of concentrate than heifers in preparation for their feedlot phase. Feed composition and nutrition composition are listed in Supplementary Table S1. Postweaning steers were transported to a contract feeder at ~1 year of age. Steers were kept in the same pen and fed a standard concentrate diet at the feedlot for fattening purposes. Thirty-four heifers remained at BRU for breeding purpose when steers moved to feedlot. Other heifers were sold at 12-month-old. Therefore, 18 months old heifers were not included in this study. Animal management is shown in Fig. 1A.

### Sample collection and processing

Fecal and blood samples were collected from MAB calves at three time points covering preweaning, postweaning, and fattening stages. Fecal samples were collected as previously described [20]. Briefly, fecal samples were taken from the rectal-anal junction (RAJ) using sterile cotton swabs. Swabs with fecal samples were placed in a 15 mL conical tube on ice and were transported to the laboratory on the same day for further processing. Each swab sample was resuspended in 2 mL of Luria-Bertani broth and 2 mL of 30% glycerol, split into four 2 mL tubes and frozen in an ultra-low freezer at −80 °C. Blood samples (10 mL per calf) were collected through the jugular by venipuncture into evacuated tubes containing lithium heparin. Two milliliters of whole-blood samples collected from preweaning calves were stored at −20 °C for genotyping analysis. Blood plasma was separated from the blood samples collected at three time points by centrifugation at 1000 × *g* for 20 min at 4 °C, and the supernatant was collected and stored at −20 °C for IgG1 level measurement.

### Measurement of body weight

Body weight (BW) was measured right after birth, as well as at the same time when fecal samples were collected at the preweaning, postweaning and fattening stages, respectively. Weight gain (WG) during preweaning (WG_preweaning_ = BW_preweaning −_ BW_newborn_), postweaning (WG_postweaning_ = BW_postweaning − _BW_preweaning_), and fattening stages (WG_fattening_ = BW_fattening − _BW_postweaning_) was calculated for further analysis.

### 16S rRNA gene sequencing and bioinformatic analysis

Fecal samples were thawed on ice and homogenized, and 500 µL of each fecal sample was used for DNA extraction using QIAamp PowerFecal DNA kit according to the manufacturer’s instructions (Qiagen, USA). The V4 region of the 16S rRNA gene was amplified, and the sequencing was conducted on the MiSeq platform (2 × 250 bp) [21].

The sequencing data was analyzed with version 2 of the Quantitative Insights into Microbial Ecology (QIIME 2) pipeline [22]. Paired-end reads were imported, and the quality of the initial bases was assessed according to the Interactive Quality Plot. The sequence quality control was conducted with the Divisive Amplicon Denoising Algorithm (DADA2) pipeline that is implemented in QIIME 2, including steps for filtering low-quality reads, denoising reads, merging the paired-end reads, and removing chimeric reads. The phylogenetic tree was generated using the align-to-tree-mafft-fasttree pipeline from the q2-phylogeny plugin of QIIME 2. The sequencing depth was normalized to 10840 sequences per sample. The Shannon index and Bray–Curtis distance were measured by the core-metrics-phylogenetic method. All amplicon sequence variants (ASVs) were classified into the bacterial taxonomy using the q2-feature-classifier plugin of QIIME 2 and the SILVA 132 database (https://www.arb-silva.de/documentation/release-132/). For each growth stage, the bacterial taxa that were present in at least 50% of animals were defined as “core bacterial taxa”. The relative abundance of bacterial taxa was log-transformed before downstream statistical analysis. For the relative abundance of certain bacterial taxa that were not present in all samples, a small numeric constant (half of the detection limit: 0.00004613) was added to all values before applying the logarithmic transformation.

### Measurement of IgG1 level in blood plasma

The IgG1 level was detected using a bovine IgG1 ELISA Quantitation set (Bethyl Laboratories, USA) according to the manufacturer’s protocol. Blood plasma was diluted in TBS-Tween to a final dilution factor of 4 × 10^4^. All dilutions were duplicated. Absorbance was read using a BioTek Synergy plate reader (BioTek Instruments, Inc., USA) at a wavelength of 450 nm. Each sample was assayed in duplicate.

### Animal genotyping

In total, 250 MAB calves were genotyped as described previously [23]. Briefly, DNA was extracted from blood samples using a QIAamp DNA mini kit according to the manufacturer’s instructions (Qiagen, USA). Genotyping was then conducted using GeneSeek Genomic Profiler F-250 at Neogen Corporation (GGP F-250, Neogen Genomics, USA). Genotypes of 221,049 SNPs were obtained for each animal. For each growth stage, individuals and genetic markers were filtered with the check.marker function from R package *GenABEL* using the following criteria: (1) Individuals and SNPs with call rate <0.95; (2) Individuals with very high autosomal heterozygosity (FDR < 1%) and very high identical by state (IBS) (FDR < 1%); (3) SNPs with minor allele frequencies (MAF) < 0.05; (4) SNPs with significant deviations from Hardy–Weinberg equilibrium (*p* < 0.05); and (5) SNPs on the Y-chromosome. After quality control, 226 out of 236 preweaning calves with 92,349 SNP markers, 176 out of 195 postweaning calves with 85,065 SNP markers, and 93 out of 105 fattening steers with 85,350 SNP markers passed all criteria and were included for further analysis.

### Statistical analyses

#### Evaluation of growth stage effect on gut microbial community

Differences in Shannon indexes among growth stages were analyzed using one-way analysis of variance (ANOVA) followed by Tukey’s HSD tests for pairwise comparison of multiple means. Differences in Bray–Curtis distances among growth stages were analyzed using a permutational multivariate analysis of variance (PERMANOVA) with the beta-group-significance command. Growth stage was set as fixed effect in the model.

#### Evaluation of breed composition effect on gut microbial community and phenotypic traits

Differences in Bray–Curtis distances among breed groups were analyzed using a permutational multivariate analysis of variance (PERMANOVA) with the beta-group-significance command. Breed group was set as fixed effect in the model. To evaluate the effect of breed composition on specific gut bacteria and IgG1 level in blood plasma, multiple linear regression models were fitted using breed composition, age, and sex as explanatory variables, and log-transformed relative abundance of core bacterial taxa or IgG1 levels as responsive variables. For weight gain, initial body weight and age intervals were also included as explanatory variables. A *p* < 0.05 was considered to be statistically significant, and 0.05 < *p* < 0.10 was considered as a tendency towards significance.

#### Genome-wide association study using a two-step mixed model-based approach

Whole-genome scans were conducted in order to measure heritability and identify specific SNPs affecting the relative abundance of core bacterial taxa at each growth stage. These analyses were performed using a two-step mixed model-based approach [24]. In the first step, the following mixed model was fitted, **y** = **Xβ** + **Zu** + **e**, where **y** is the vector of log_10_ transformed relative abundance of core bacteria, **β** is the vector of fixed effects, such as age and sex, **u** is the vector of random animal effects, and **e** is the vector of random residuals. Matrices **X** and **Z** are incidence matrices relating phenotypic records to fixed and animal effects, respectively. Random vectors **u** and **e** were assumed to be normally distributed as **u** ~ *N* (**0, G**${\mathbf{\sigma }}_{\boldsymbol{u}}^2$) and **e** ~ *N* (**0, I**${\mathbf{\sigma }}_{\boldsymbol{e}}^2$), where **G** is the genomic relationship matrix, and **I** is an identity matrix. In the second step, the following model was fitted for every SNP, **y** = **Xβ** + **X**_**SNP**_**β**_**SNP**_ + **ε**, where **X** and **β** are defined as in the model used in step 1, **X**_**SNP**_ is an incidence matrix relating phenotypic records to number of reference alleles (0, 1, 2), β_SNP_ is the regression coefficient for the SNP, and ε is a random vector assumed to be multivariate normal with mean equal to zero and variance equal to **ZGZ**^**T**^$\sigma _u^2$ + **I**$\sigma _e^2$, where **Z, G, I**, $\sigma _u^2$, and $\sigma _e^2$ are as defined for the model in step 1. These analyses were conducted using R package *MixABEL*. The genomic control procedure was applied to correct for a possible inflation using the function VIFGS implemented in the R package GenABEL. The *p*-values were adjusted for multiple testing using the Benjamini–Hochberg method. Associations with FDR < 0.05 were considered significant.

#### Co-occurrence network analysis

To predict bacteria–bacteria interactions in the gut microbial community at each stage, co-occurrence patterns of core bacterial genera within each stage (bacterial genera present in at least 50% of samples at preweaning, postweaning, and fattening stages, respectively) were evaluated in the network interface using pairwise Spearman’s rank correlations (*r*_s_) based on relative bacterial abundance [25]. The Spearman’s rank correlations were analyzed using the *Hmisc* package of the R software. A significant rank correlation between two genera (*r*_s_ > 0.25 or *r*_s_ < −0.25, FDR-adjusted *p* < 0.001) was considered to be a co-occurrence event. The network was visualized using the Force Atlas algorithm in the interactive platform Gephi (http://gephi.org). Network nodes represent different core bacterial genera, and edges indicate significant correlations between nodes. The size of the nodes reflects the degree of connection, and the thickness of the edges indicated the strength of the correlation. The most densely connected node in each network was defined as the “hub”.

#### Potential contribution of gut microbiota to phenotypic traits

The potential effect of gut microbiota on weight gain and IgG1 levels in blood plasma at each growth stage was first evaluated using microbiability, defined as the proportion of total phenotypic variance explained by the microbiome [26, 27]. Briefly, the following linear mixed model was fitted: **y** = **Xβ** + **Wm** + **e**, where **y** is the vector of phenotypic traits, **β** is the vector of fixed effects, **m** is the vector of random animal effects, and e is the vector of random residuals. Matrices **X** and **W** are incidence matrices relating phenotypic records to fixed and animal effects, respectively. Random vectors m and e were assumed to be normally distributed as **m** ~ N (**0**, **M**$\sigma _m^2$) and **e** ~ N (**0**, **I**$\sigma _e^2$), where **M** is the microbial relationship matrix, and **I** is an identity matrix. The microbial relationship matrix **M** was constructed as follows, **M** = **OO**^T^/n, where **O** is the matrix of log-transformed relative abundance of amplicon sequence variants (ASVs), and n is the total number of different ASVs. Only ASVs that were present in at least 50% of the animals were considered. Before log transformation, a small numeric constant (half of the detection limit: 0.00004613) was added to all values. The **O** matrix was scaled and centered (mean = 0, SD = 1) before calculating the **M** matrix. In this scenario, $\sigma _m^2/\left( {\sigma _m^2 + \sigma _e^2} \right)$ is considered the microbiability. The potential contribution of each core bacterial taxa to animal phenotype at each growth stage was also evaluated using the following linear mixed model, **y** = **Xβ** + **X**_**bac**_**β**_**bac**_ + **Zu** + **e**, where **y** is the vector of phenotypic values (either weight gain or IgG1 levels), **β** is the vector of fixed effects, **u** and **e** are the vectors of animal and residual effects, respectively, with **u** ~ N (**0**, **G**$\sigma _u^2$), and **e** ~ N (**0**, **I**$\sigma _e^2$), **X**_**bac**_ represents the log_10_ transformed relative abundance of the core bacteria under study and **β**_**bac**_ is the regression coefficient, i.e., the effect of core bacteria on the phenotype. This model allows us to evaluate the effect of core bacteria on the phenotype, controlling simultaneously for the animal effect (population structure). Preliminary, we also evaluated the effect of core bacteria but without considering the animal effect. In both analyses, the significance of **β**_**bac**_ was evaluated using a *t*-test.

#### Associations between breed composition and MAF of SNPs located in SCFAs receptors

The associations between genotype of SNPs located in SCFAs receptors (GPR41, GPR43, and GPR109A) and breed composition were evaluated using Spearman correlation. Genotypes were coded as 0/1/2 for genotypes aa/aA/AA. Significant associations between SNPs and breed composition were considered with a *p* < 0.05.

## Results

### Dynamics of the gut microbiota in different stages of growth

To understand the influence of host genetics on gut microbiota development, we bred calves using the multibreed Angus-Brahman (MAB) population and confirmed gradual change of genetic composition of the bovine model by measuring genetic distance and physiological parameters [12]. In this longitudinal study, we evaluated prolonged host genetic effects on gut microbiota in different growth stages using the MAB population throughout life. As shown in the schematic diagram (Fig. 1A), the naturally delivered newborn MAB calves (*n* = 279) were raised together with their dams on pasture until calves were separated on the basis of sex at weaning. Three months after weaning, steers were transported to a feedlot to be fed with high-concentrate diet for fattening.

Gut microbiota of the MAB herd was evaluated at preweaning (~3 months old, *n* = 239), postweaning (~12 months old, *n* = 195), and fattening stages (~18 months old, *n* = 105). An average of 32,001 ± 434 (mean ± SEM) OTUs per sample were generated for bacteria from a total of 26,160,876 raw reads obtained from 538 fecal samples (Supplementary Table S2). The bacterial diversity reflected by Shannon index increased after weaning, however, it decreased after calves were transferred to the feedlot (Fig. 1B). Gut microbiota structure was significantly different in the three growth stages as shown in the PCoA plot based on the Bray–Curtis distance (*p* = 0.001) (Fig. 1C). Notably, the gut microbiota of preweaning and fattening calves showed larger variation within the group than that of postweaning calves, indicating that gut microbiota is dynamic during growth rather than synchronized.

The dominant phyla were *Firmicutes* and *Bacteroidetes*, accounting for >85% of the total bacteria in the three stages (Supplementary Fig. 1). *Proteobacteria* was the third most abundant phylum in preweaning and fattening stages (6.5%) but showed relatively low abundance in postweaning stage (2%). At family level, *Ruminococcaceae* was the most abundant in both preweaning (23.6%) and postweaning (34.5%) stages, followed by *Prevotellaceae*, *Bacteroidaceae*, *Lachnospiraceae* and *Rikenellaceae*, each representing >5% of total bacteria. At fattening stages, however, *Prevotellaceae* became the most dominant bacteria (24.2%), followed by *Ruminococcaceae* (21.5%), *Lachnospiraceae* (10.3%) and *Bacteroidaceae* (7.2%) (Fig. 1D).

### Host genetics shapes the gut microbiota throughout life

**Fig. 2:**
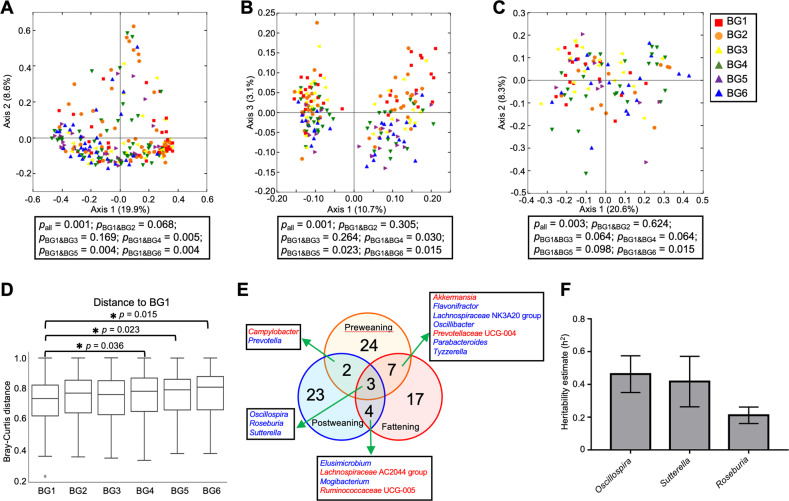
Host genetics impacts gut microbiota in different growth stages. **A**–**C** PCoA plots of Bray–Curtis distances comparing gut microbiota composition among the six multibreed groups (BGs) in the preweaning (**A**), postweaning (**B**), and fattening (**C**) stages, respectively. **D** Boxplots of Bray–Curtis distances between microbial communities obtained when comparing individuals within BG1 and those within other five BGs. Gut microbiota data from the three growth stages were combined to analyze overall breed composition effects. Differences in Bray–Curtis distances among BGs were analyzed using PERMANOVA. **E** Venn diagram showing number of core bacterial genera that are associated with breed composition at each stage and shared breed-associated core bacterial genera from the three growth stages. **F** Heritability estimates (*h*^2^) of three bacterial genera that are consistently associated with breed composition at the three growth stages. Results are presented as mean of heritability across three stages with standard errors.

To evaluate the role of host genetics in shaping gut microbiota throughout life, we first investigated whether breed composition has prolonged effects on the gut microbiota in different growth stages. Environmental conditions including management and diet were identical across breed groups (BGs) at each stage for concise evaluation of host genetic effect. Gut microbiota structure was significantly influenced by breed composition in preweaning (Fig. 2A), postweaning (Fig. 2B), and fattening (Fig. 2C) stages, showing greater dissimilarity with increasing genetic distance, regardless of growth stage. Although individual variation in postweaning calves was significantly less than other stages (Fig. 1C), breed composition effects on microbiota structure, captured by PCoA Axis 3, were still significant (Fig. 2B). PCoA Axis 3 separated the microbiota structure among breed groups in both heifers and steers (Fig. 2B). PCoA Axis 1 separated the microbiota structure due to the confounding effects of sex and diets in postweaning calves (Supplementary Fig. 2B), but not in preweaning calves (Supplementary Fig. 2A). Breed composition effects on gut microbiota, analyzed with combined microbiota of all three stages together, showed that gut microbiota structure of calves was significantly different among the six BGs (Fig. 2D, *p* = 0.001). The greatest difference in microbiota structure was observed between BG1 and BG6 (*p* = 0.015), the calves of which had the greatest genetic distances; this indicates that the effects of host genetics are not specific to certain growth stages, but are universal throughout life.

To identify specific bacterial genera affected by breed composition, associations between breed composition and the log_10_ transformed relative abundance of core bacterial taxa were evaluated using multiple linear regression models that included the explanatory variables of age, sex, and breed composition. At the genus level, the relative abundances of 36 (52.2%) out of 69, 32 (40%) out of 80, and 31 (37.3%) out of 83 core bacterial genera were significantly associated or showed tendency with breed composition in preweaning, postweaning and fattening calves, respectively (Fig. 2E, Supplementary Fig. 3, and Supplementary Tables S3–S5, *p* < 0.1). Among the bacterial genera, the relative abundance of *Oscillospira*, *Roseburia* and *Sutterella* showed positive associations with Brahman composition throughout life (Fig. 2E). Interestingly, *Oscillospira* (*h*^2^ = 0.46) and *Sutterella* (*h*^2^ = 0.42) showed relatively high heritability estimates (Fig. 2F), which indicates their colonization is dramatically influenced by host genetics, while *Roseburia* (*h*^2^ = 0.21) seems to be more susceptible to environmental conditions.

**Fig. 3:**
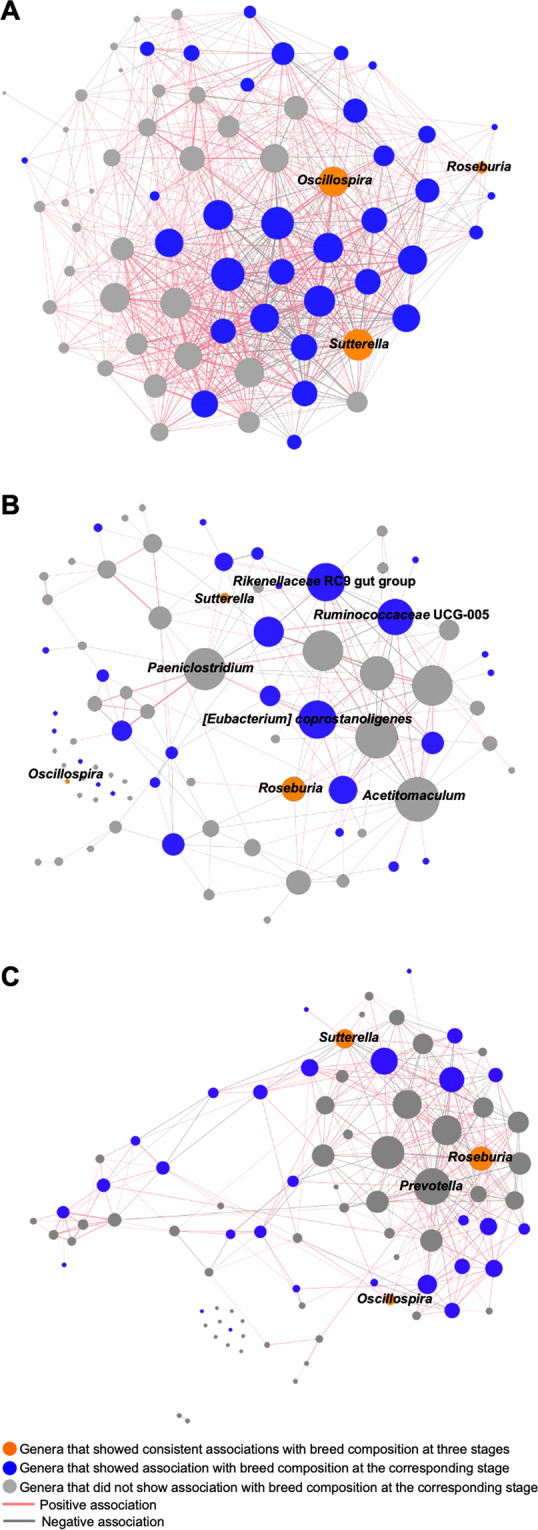
Co-occurrence network of core gut microbial genera. **A**–**C** Co-occurrence networks predicting the bacteria–bacteria interactions among core bacterial genera (present in at least 50% of the samples) in the preweaning (**A**), postweaning (**B**), and fattening (**C**) stages, respectively. Connections were detected based on Spearman’s rank correlations (*r*_s_ > 0.25 or *r*_s_ < −0.25, FDR-adjusted *p* < 0.001). Dot sizes represent number of connections with other taxa. Thickness of edges represent strength of relatedness. Dot color represents the relationship between relative abundance of bacterial genera and breed composition. Edge color represents either positive or negative associations between bacteria.

As bacteria–bacteria interactions are key modulators to shape the gut microbiota, we further evaluated whether host genetics, especially breed composition, affects bacteria–bacteria interactions in different growth phases using a bacterial co-occurrence networks analysis. A Spearman’s correlation coefficient > 0.25 or < −0.25 and an adjusted *p* < 0.001 were considered indicative of a bacterial connection. As shown in Fig. 3, the bacterial network structure changed dynamically based on growth stages, which is likely due to variations in environmental conditions, age, and sample size among stages. Dense bacteria–bacteria interactions were observed in preweaning calves (Fig. 3A). Multiple breed-associated bacteria (presented in blue and orange nodes) that had significant associations with breed composition, were identified as hub bacteria in preweaning stage, including *Oscillospira and Sutterella*. However, non-breed-associated bacteria, *Acetitomaculum* and *Paeniclostridium*, were identified as hub bacteria in the postweaning stage (Fig. 3B). These two bacteria showed different relative abundances between heifers and steers (Supplementary Table S4), which suggests that the effects of sex and diets mainly drives the bacteria–bacteria interactions at the postweaning stage. Finally, in the fattening stage, *Prevotella*, the relative abundance of which dramatically increased after calves were moved to a feedlot, had the largest number of interactions in the network (Fig. 3C).

### The gut microbiota is linked to animal growth and immunity throughout life

**Fig. 4:**
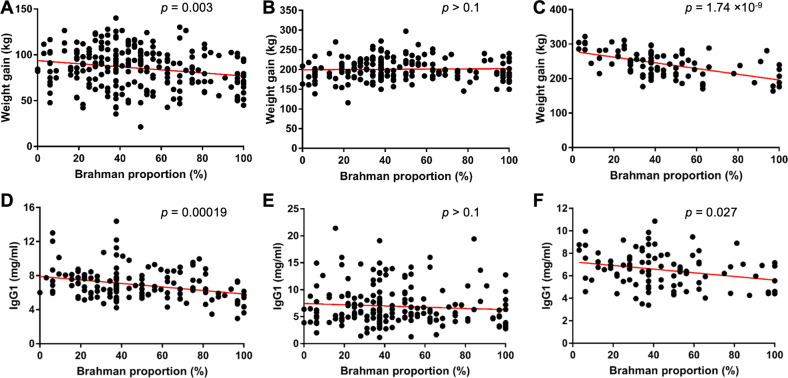
Associations between breed composition, animal weight gain, and IgG1 levels in blood plasma. **A**–**C** Associations between breed composition and weight gain during the preweaning (**A**), postweaning (**B**), and fattening (**C**) stages, respectively. These associations were analyzed using multiple linear regression models, with initial body weight, age, sex, and breed composition as explanatory variables, and weight gain as response variables. **D–F** Associations between breed composition and IgG1 levels in blood plasma during the preweaning (**D**), postweaning (**E**), and fattening (**F**) stages, respectively. These associations were analyzed using multiple linear regression models, with age, sex, breed composition as explanatory variable and IgG1 levels in blood plasma as response variable.

To understand if microbiota in the GI tract may have an effect upon phenotypic traits throughout life, we focused on animal growth rate and immunity. It has been suggested that hindgut microbial fermentation fermentation plays a critical role in digestion and energy harvest in preweaning calves when the rumen is not fully developed [28]. Later, the intestinal tract accounts for only 10% of the total energy harvest when the rumen is fully developed [28]. Moreover, Brahman (*Bos indicus*) are more resistant to parasite infection in tropical regions than Angus (*Bos taurus*) [29, 30], which indicates underlying differences in their immune functions. Therefore, we hypothesized that variations in microbiota composition mediated by host genetics may also modulate animal growth and immunity throughout life. To evaluate our hypothesis, we measured associations between gut microbiota, animal growth, and immunity. Associations between weight gain and breed composition were detected in the preweaning (Fig. 4A) and fattening (Fig. 4C) stages. IgG1 levels in blood plasma were negatively associated with Brahman proportion in the preweaning (Fig. 4D) and fattening (Fig. 4F) stages. However, these associations were not observed in the postweaning stage (Fig. 4B, E). Animal growth rates and IgG1 levels were significantly greater in steers than in heifers during the preweaning and postweaning stages (Supplementary Fig. 4), which suggests that sex and diet were predominant factors.

**Fig. 5:**
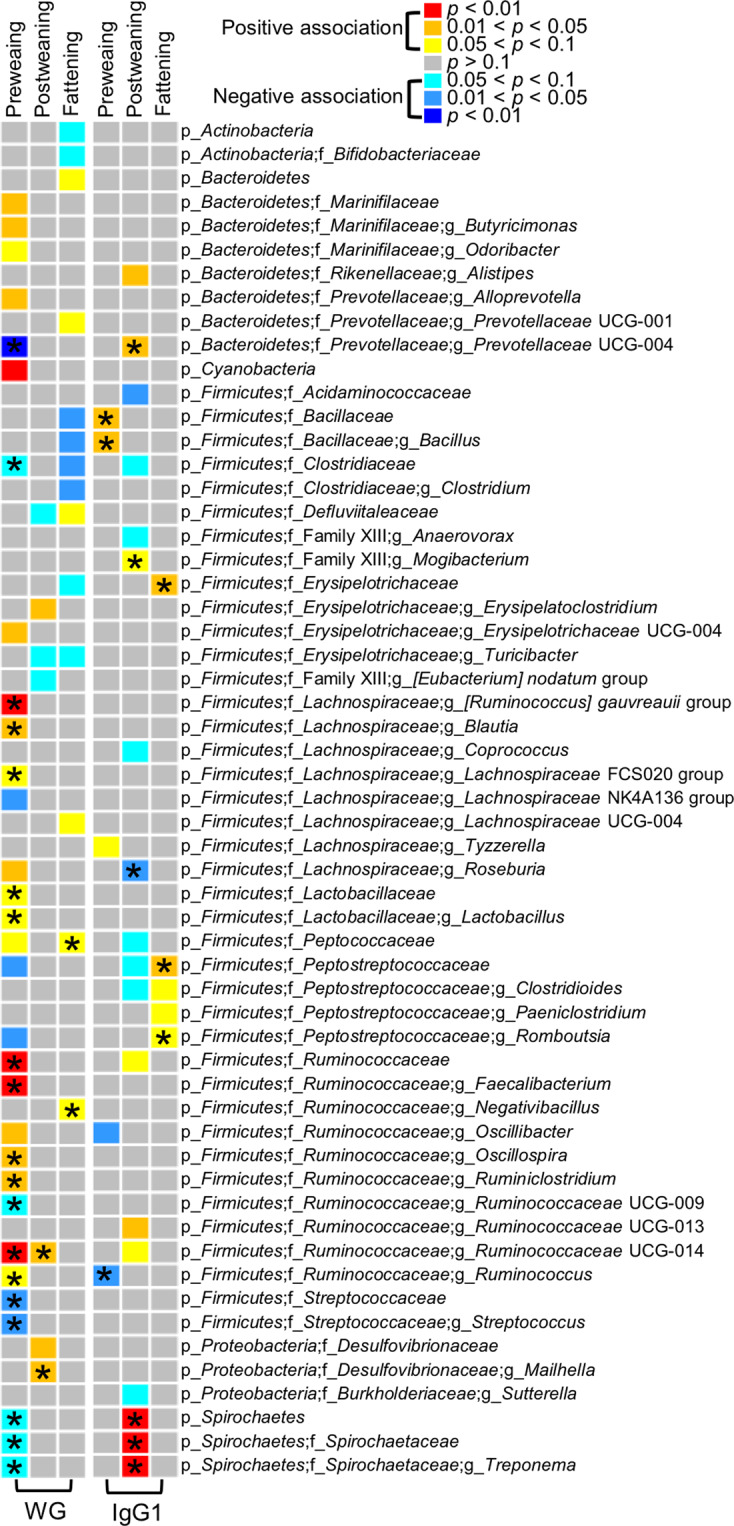
Heatmap showing associations between core bacterial taxa (phylum, family, and genus), and animal weight gain, and IgG1 levels in blood plasma. Associations between weight gain and core bacteria were analyzed using multiple linear regressions, with initial body weight, age, breed composition, sex, and the relative abundance of bacteria as explanatory variables. Associations between IgG1 levels in blood plasma and core bacteria were also analyzed using multiple linear regressions. The asterisks denote associations that were significant or had tendency when the analysis was performed using an animal mixed model (controlling the animal effect).

The average microbiability (proportion of total phenotypic variance explained by the microbiome) for weight gain and IgG1 levels throughout life was 0.106 and 0.100, respectively (Supplementary Table S6). To identify specific bacterial genera that potentially affect weight gain and immunity throughout the animal’s growth stages, we analyzed associations between the relative abundance of bacteria and either weight gain or immunity. We first used a multiple linear regression model that included the initial body weight, age intervals, breed composition estimated by pedigree, sex and relative abundance of bacteria as the explanatory variables, and weight gain as the response variable. At the genus level, we identified 21 out of 69, 5 out of 80, and 6 out of 83 core bacterial genera that were associated with weight gain in the preweaning, postweaning and fattening stages, respectively (Fig. 5 and Supplementary Tables S7–S9). More bacterial taxa were associated with animal growth in the preweaning stage, especially butyrate-producing bacteria, such as *Butyricimonas*, *[Ruminococcus] gauvreauii* group*, Blautia*, *Roseburia, Faecalibacterium*, *Oscillibacter*, *Oscillospira*, and *Ruminiclostridium*, which all showed positive associations with weight gain. Out of the 31 core bacterial genera that showed associations with weight gain when breed composition was used as the explanatory variables, 15 remained significant or had tendency when the statistical model accounted for population structure, i.e., animal effect (Fig. 5 and Supplementary Tables S7–S9). Most significant associations were detected during the preweaning stage.

IgG1 levels in blood plasma were associated with four, 11, and three bacterial genera in the preweaning, postweaning and fattening stages, respectively (Fig. 5 and Supplementary Tables S10–S12); this association was detected using the first model, that included the age, sex, breed composition and the relative abundance of bacteria as the explanatory variables. Among the 18 genera, 7 remained significant or had tendency when using the second (animal) model. High blood plasma IgG1 levels were associated with *Spirochaetes*, especially the *Treponema* genus, which are invasive bacteria that cause cattle enteritis. Interestingly, some butyrate-producing bacteria *Roseburia, Oscillibacter and Ruminococcus* showed associations with greater weight gain and lower IgG1 levels, which suggests that energy harvest and immunity are inversely linked by these bacteria.

### Genomic scan identified host SNPs associated with bacteria that potentially contribute to weight gain and immunity

**Fig. 6:**
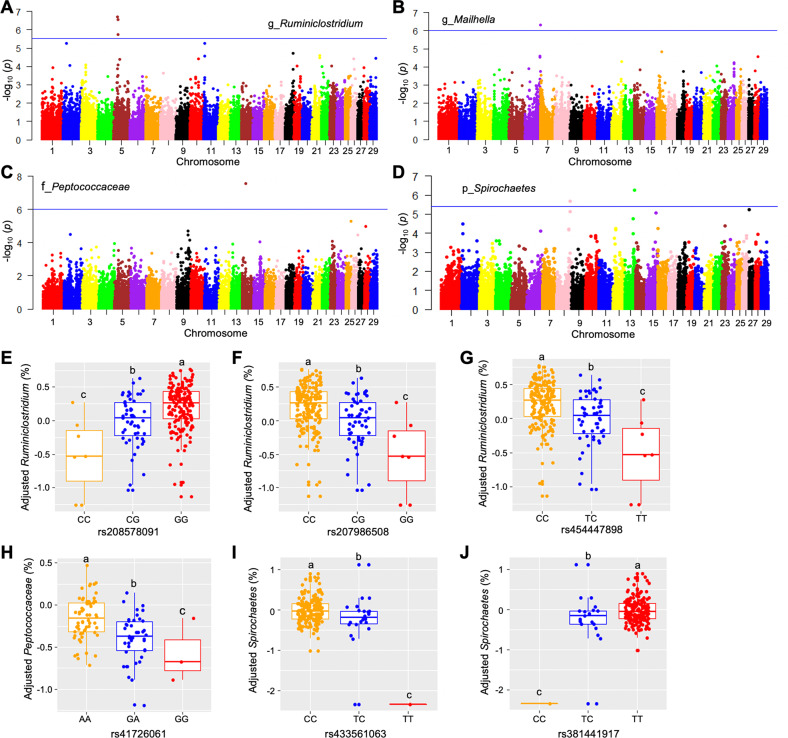
Associations between host SNPs and phenotype-related bacteria. **A**–**D** Manhattan plots showing SNP significance across the entire bovine genome. SNPs were associated with log-transformed relative abundance of *Ruminiclostridium* (**A**), *Mailhella* (**B**), *Peptococcaceae* (**C**), and *Spirochaetes* (**D**) using a two-step mixed model-based approach. The horizontal lines represent FDR-adjusted genome-wide significance (*p* < 0.05). **E**–**J** Adjusted relative abundance of weight gain and blood plasma IgG1 level-associated bacteria among calves with different genotypes of SNPs located in genes involved in metabolic or immune pathways. In each plot, different superscripts denote significant differences based on one-way ANOVA followed by Tukey’s multiple comparison tests (*p* < 0.05).

To further investigate whether weight gain and immunity-associated bacteria were influenced by variations in the host genome, genomic scans were conducted to identify specific SNPs that were associated with the relative abundance of these bacteria. A total of ten SNPs with known reference SNP (rs) ID located in or near protein-coding genes were detected as significantly associated with three weight-gain-associated bacteria and one IgG1-associated bacteria (Fig. 6A-D and Supplementary Table S13).

Among the ten SNPs, six SNPs are located in genes associated with metabolism, immunity or GI tract development (Supplementary Table S13). As shown in Fig. 6, among weight-gain-associated bacteria, *Ruminiclostridium* is associated with two SNPs (rs208578091 and rs207986508) located in *ZNF641*, which encodes Zinc finger protein 641, that participates in herpes simplex virus 1 infection, and with one SNP (rs454447898) in phosphofructokinase gene *PFKM*, which catalyzes the phosphorylation of fructose-6-phosphate to fructose-1,6-bisphosphate (Supplementary Table S13). *Peptococcaceae* is associated with one SNP (rs41726061) in *NSMAF*, encoding a neutral sphingomyelinase activation-associated factor required for TNF-mediated activation of neutral sphingomyelinase, and may play a role in inflammation (Supplementary Table S13). Differences among log_10_ transformed relative abundance of weight-gain-associated bacteria from calves with different genotypes for these SNPs were compared (Fig. 6E–H). For IgG1-associated bacteria, *Spirochaetes* was associated with one SNP (rs433561063) located in catenin alpha-like 1 (*CTNNAL1*) gene, which is related to inflammation, and one SNP (rs381441917) located in erythrocyte membrane protein band 4.1 like 4B (*EPB41L4B*) gene, which is involved in regulation of the tight junction of epithelial cells and plays a role in wound healing (Supplementary Table S13). Differences among log_10_ transformed relative abundance of IgG1-associated bacteria from calves of different genotypes for these SNPs were compared (Fig. 6I-J).

### Identification of SNPs in genes associated with butyrate-producing bacteria and SCFA receptors

SCFAs, such as acetate, propionate, and butyrate, are critical for both animal growth and epithelial immunity [31]. In particular, butyrate is a main energy source of colonocytes [32]. As the relative abundances of butyrate-producing bacteria, *Oscillospira* and *Roseburia*, were associated with breed composition in all three growth stages (Fig. 2E), we further investigated to understand if the relative abundance of these bacteria were associated with variations in the host genome. Indeed, we identified nine SNPs that were significantly associated with the relative abundances of these bacteria in the three growth stages (Supplementary Table S14). Six SNPs were associated with the relative abundance of *Roseburia*. These SNPs are located in genes encoding COMM Domain Containing 5 (rs458474702), OTU Deubiquitinase (rs211515678), interferon-stimulated gene 12b protein (rs453206783), serpin family A member 12 (rs210993880), located in a gene encoding Rho GTPase Activating Protein 22 (rs444932446), and LDL Receptor-Related Protein 5 (rs42190891). Three SNPs were associated with the relative abundance of *Oscillospira*. These SNPs were located in genes encoding collagen type VII alpha 1 chain (rs110729066) or dermatan sulfate epimerase like protein (rs137443102 and rs378568110). Taken together, we observed associations between host SNPs and the relative abundance of butyrate-producing bacteria throughout life, indicating that host genetics may affect weight gain in animals partially through regulating gut microbiota.

**Fig. 7:**
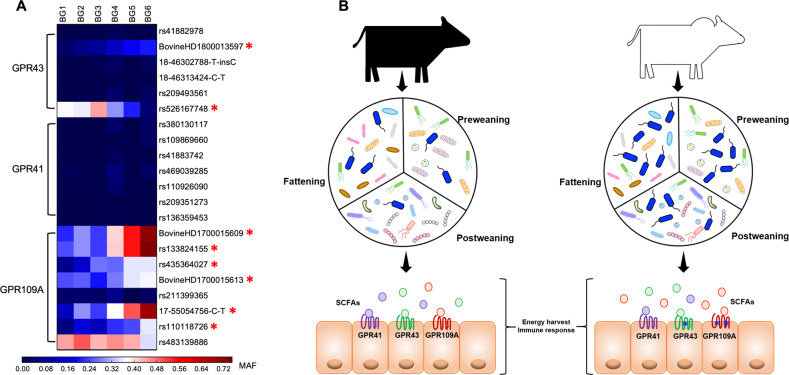
Associations between host SNPs in SCFA receptor genes and breed composition. **A** Heatmap showing the associations between SNPs located in SCFA receptor genes and breed composition based on Spearman’s rank correlation. The asterisks indicate significant associations between SNP genotype and breed composition (*p* < 0.05). **B** Graphical summary showing the prolonged host genetic effects on gut microbiota and SCFA receptors contributing to animal growth and immunity. Host genetics influences the gut microbiota across life. A significant portion of gut microbiota is affected by breed composition differently depending on the animal’s growth stage. Butyrate-producing bacteria such as *Roseburia* and *Oscillospira* are enriched in calves with more Brahman than Angus proportion during growth. Genotypes of SCFA receptors, especially *GPR43* and *GPR109A* (marked with asterisks), also vary across genetic groups, which also may contribute to the differences in growth and immunity among calves by responding to the SCFAs and downstream regulation pathways.

SCFAs, in particular butyrate, promote anti-inflammation and enhance intestinal epithelial barrier function through binding and activating SCFAs receptors. The G protein-coupled receptors *GPR109A*, *GPR41*, and *GPR43* are three major and well-known SCFAs receptors genes [33–36]. In order to evaluate whether there was an association between genetic markers in SCFAs receptors and breed composition, we analyzed the minor allele frequency (MAF) of SNPs located in genes *GPR41*, *GPR43* and *GPR109A* among BGs. Interestingly, two out of 6 SNPs located in *GPR43* and six out of 8 SNPs located in *GPR109A* showed significant differences in MAF among breed composition groups (Fig. 7A and Supplementary Table S15). Taken together, these findings indicate that genetic variations in SCFAs receptors, especially in *GPR109A* and *GPR43*, may contribute to the observed differences in immune response and energy harvest among the MAB population (Fig. 7B).

## Discussion

In this study, we show that gut microbiota structure is influenced by host genetics throughout life. The structure of the gut microbiota was found to change within the GI tract of calves during growth in the preweaning, postweaning and fattening stages. However, we also found that this microbiota structural change is also associated with a host’s gradual change of breed composition, which we interpret as strong evidence that host genetics influences the gut microbiota structure throughout life. Moreover, we identified specific bacterial genera that potentially contribute to energy harvest and immunity at each life stage. The differences in the relative abundances of several growth and immunity-associated bacteria were closely linked to host SNPs located in genes involved in energy metabolism and immunity. These findings demonstrate that host genetics modulates its microbiota structure, and this modulation lasts across life.

The gut microbiota develops as the animals grow, and differences in the gut microbial community structure are driven by intrinsic and extrinsic factors such as age, sex, host genetics, environmental conditions, diet, and geographical location [3–6, 37]. It was recently reported that environmental factors, such as household sharing, have a predominant role in shaping human microbiota [8]. Consistently, in this cohort study, we also observed dynamic changes in gut microbiota structure in calves at different growth stages. These dynamic changes were probably coupled with changes in diet, age, sex, and housing, which are known to have a strong influence in shaping the gut microbiota. As shown in Fig. 1B, bacterial diversity and microbiota structure changed significantly during growth. The gut microbiota changed significantly when calves were moved to a feedlot where they were fed with high-concentrate diets. *Prevotellaceae* took the place of *Ruminococcaceae* as the most dominant bacterial family in the fattening stage, consistent with the previous study where cattle were fed with grains instead of forage [38]. *Clostridiaceaea* increased sharply while *Rikenellaceae* decreased in fattening calves. This pattern was detected in the hindgut of swine fed with high level of protein in diet as well [39].

However, regardless of changes in the gut microbiota structure during the growth stages and changes in diet and living environment, the impact of host genetics on gut microbiota composition remained consistent across multibreed groups. We found the largest differences in gut microbiota structure between BG1 (mostly Angus) and BG6 (mostly Brahman), which have the greatest genetic distance. In addition, as shown in Fig. 2E, we identified core bacterial genera that showed associations with breed composition depending on growth stage. More than one-third of the total core bacterial genera were associated with breed composition in each growth stage, which indicates that host genetics has prolonged effects rather than temporal effects. Of the breed-associated genera, three bacterial genera including *Oscillospira*, *Roseburia* and *Sutterella* showed consistent associations with breed composition in all three growth stages, with *Oscillospira* and *Sutterella* also had high heritability throughout life. Interestingly, these three bacteria have been reported to be heritable in human gut microbiota [19, 31, 40]. Furthermore, although these bacteria are commensal bacteria in the GI tract of cattle, their functions have been mainly investigated in human studies. Both *Oscillospira* spp. and *Roseburia* spp. are butyrate-producing bacteria and are associated with reduced incidence of inflammatory disease due to their anti-inflammatory properties [41, 42]. In contrast, *Sutterella* spp. have been reported to have mild pro-inflammatory capacity in the human GI tract and are associated with autism spectrum disorders especially in children [43, 44]. *Roseburia* spp. utilize a variety of dietary polysaccharide substrates, while *Oscillospira* spp. are able to utilize host-derived glycans (e.g., fucose, sialic acids, and glucuronic acid), which partly explains the association with leanness [45]. Interestingly, we identified two significant SNPs that are located in the *DSEL* gene, which is involved in glycosaminoglycan metabolism pathways [46]. These two SNPs were associated with the relative abundance of *Oscillospira* at all three growth stages, suggesting that genetic variation in *DSEL* may result in different abilities to utilize host-derived glycans among the MAB population.

Regarding the potential impact of gut microbiota on animal growth and health, we discovered that more bacteria in preweaning calves were associated with weight gain than in the postweaning and fattening growth stages, which is consistent with the critical role of hindgut fermentation when the rumen is not fully developed [28]. Further, several bacteria that showed positive or negative associations with weight gain, had negative associations with blood IgG1 levels. Thus, these bacteria could be closely linked to energy harvest and immune function. For example, both *Treponema* and *Bacillus* genera that include opportunistic pathogenic species [47, 48], cause infections that may result in reduced calf growth. In contrast, butyrate-producing bacteria *Roseburia*, *Oscillibacter* and *Ruminococcus*, as well as the potential butyrate-producer *Peptococcaceae* likely suppress inflammation and promote calf growth by butyrate production in the GI tract [49]. We also found genotypes of SCFA receptors, especially *GPR109A*, a butyrate receptor [50], which were strongly associated with breed composition, and that might lead to the variation in response to SCFAs across breed groups and further impact the metabolism and immune function mediated by SCFAs and their receptors [51, 52].

Moreover, we identified SNPs that are located in the genes, which are involved in metabolism and immunity that showed significant associations with weight gain and IgG1 level-related bacteria. The weight-gain-related bacteria *Ruminiclostridium*, which mainly produces ethanol, acetate and lactate by metabolizing cellulose and hemicellulosic polysaccharides, was associated with one SNP located in the *PFKM* gene, encoding phosphofructokinase, a critical enzyme involved in the glycolytic pathway [53]. The relative abundance of invasive pathogenic bacteria *Spirochaetes*, which causes enteritis in cattle [54], was associated with high IgG1 level and was also associated with one SNP located in the *CTNNAL1* gene, catenin alpha-like 1 protein, which affects the NF-kB and MAPK pathways [55]. Recently, Li et al. [11] identified six SNPs associated with Spirochaetes in rumen microbiota, including two SNPs in the optineurin (*OPTN*) genes, a negative regulator of NF-kB interacting with TNF-α to mediate inflammation [56]. These results suggest that cattle immune genes may determine the *Spirochaetes* prevalence in both the rumen and hindgut. Taken together, there is strong evidence that host genetics exerts effects on modulation of specific gut microorganisms that may further influence host growth and health.

This study mainly assessed host genetic effects on gut microbiota using a multibreed beef cattle cohort in the three different growth stages. Although the gut microbiota structure was distinct among the growth stages, we found that the gut microbiota was influenced by host genetics throughout life. The current study simultaneously accessed the potential contribution of gut microbiota on cattle growth and immunology at different growth stages and extended to identify specific SNPs that are associated with the phenotypic-related bacteria. As the study was conducted using only one generation of multibreed cattle, future studies including more generations of this unique cattle population and homogenous purebred population will provide further support of our current findings. The role of some candidate bacteria, especially those persistently associated with breed composition and specific genetic markers, should be further studied. In summary, our study provides strong evidence that host genetics modulates gut microbiota composition, and our findings also contribute to the understanding of how the gut microbiota develops.

## Supplementary information

Supplementary Figures

Supplementary Tables

## Data Availability

The 16S rRNA gene sequencing data generated and analyzed during the current study are available in the NCBI primary data archive (PDA) with accession number SRP115548.
