# Supplementary material for: Host genetics exerts lifelong effects upon hindgut microbiota and its association with bovine growth and immunity
Source: ISME J. 2021 Mar 1;15(8):2306–21. doi: 10.1038/s41396-021-00925-x (PMC8319427; doi:10.1038/s41396-021-00925-x)
Supplement: Supplementary file 1 — Supplementary Figures [file 41396_2021_925_MOESM1_ESM.docx]

Supplementary information for

**Host genetics exerts lifelong effects upon hindgut microbiota and**

**its association with bovine growth and immunity**

Peixin Fan^1, 2^, Corwin D. Nelson^2^, J. Danny Driver^2^, Mauricio A. Elzo^2^, Francisco Peñagaricano^2,3^ and Kwangcheol C. Jeong^1, 2*^

^1^Emerging Pathogens Institute, University of Florida, Gainesville, FL 32611 USA

^2^Department of Animal Sciences, University of Florida, Gainesville, FL 32611 USA

^3^Department of Animal and Dairy Sciences, University of Wisconsin-Madison, Madison, WI 53706 USA

Running Title: Lifelong host genetic effects on gut microbiota

Key words:

Host genetics, gut microbiota, growth, age

*****Corresponding author

Kwangcheol C. Jeong, PhD

Associate Professor of Microbiology

Address: 2055 Mowry Rd, Gainesville, FL 32611 USA

Email: kcjeong@ufl.edu

Phone: 1-352-294-5376

**Supplementary figures**


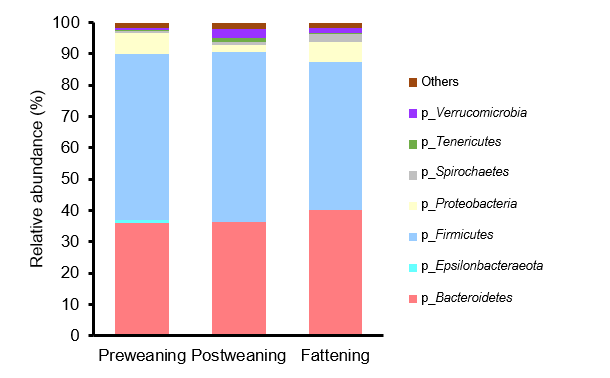


**Supplementary Figure 1. Distribution of abundant bacterial phyla (relative abundance > 1%) across different stages.**

**
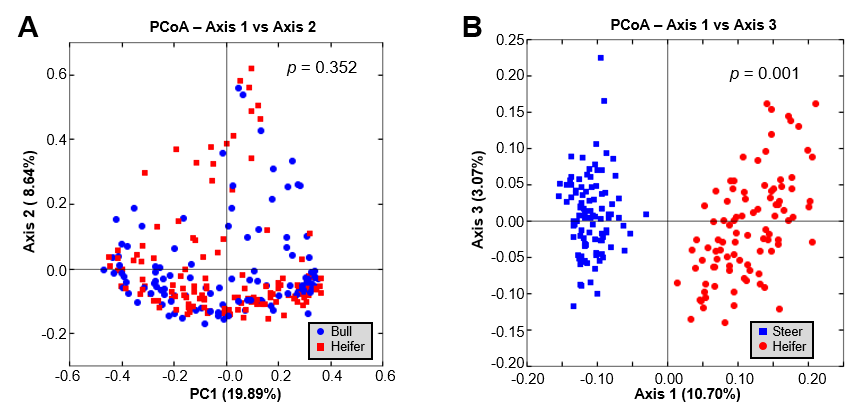
**

**Supplementary Figure 2. Differences in gut microbiota structure between heifers and bull (or steers) during preweaning and postweaning stages.** (A) PCoA plot of Bray-Curtis distance comparing gut microbiota structure between heifers and bulls during the preweaning stage. (B) PCoA plot of Bray-Curtis distance comparing gut microbiota structure between heifers and steers during the postweaning stage. The statistical difference in Bray-Curtis distance was accessed using permutational multivariate analysis of variance (PERMANOVA).


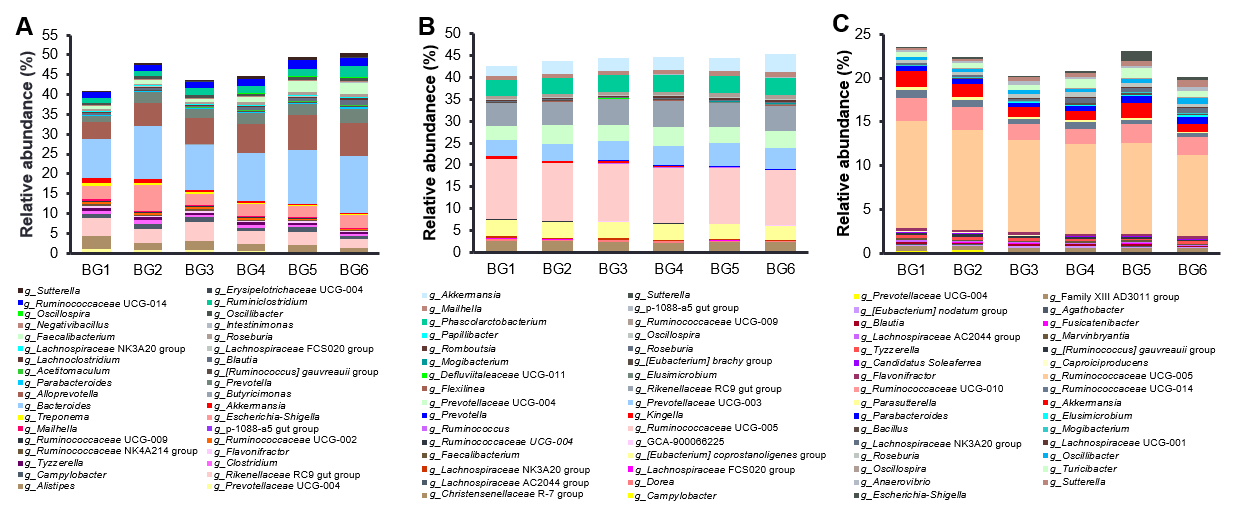


**Supplementary Figure 3. Relative abundance of bacteria that showed linear association with breed composition at different growth stages.** (A) Distribution of bacteria associated with breed composition at the preweaning stage. (B) Distribution of bacteria associated with breed composition at the postweaning stage. (C) Distribution of bacteria associated with breed composition at the fattening stage.


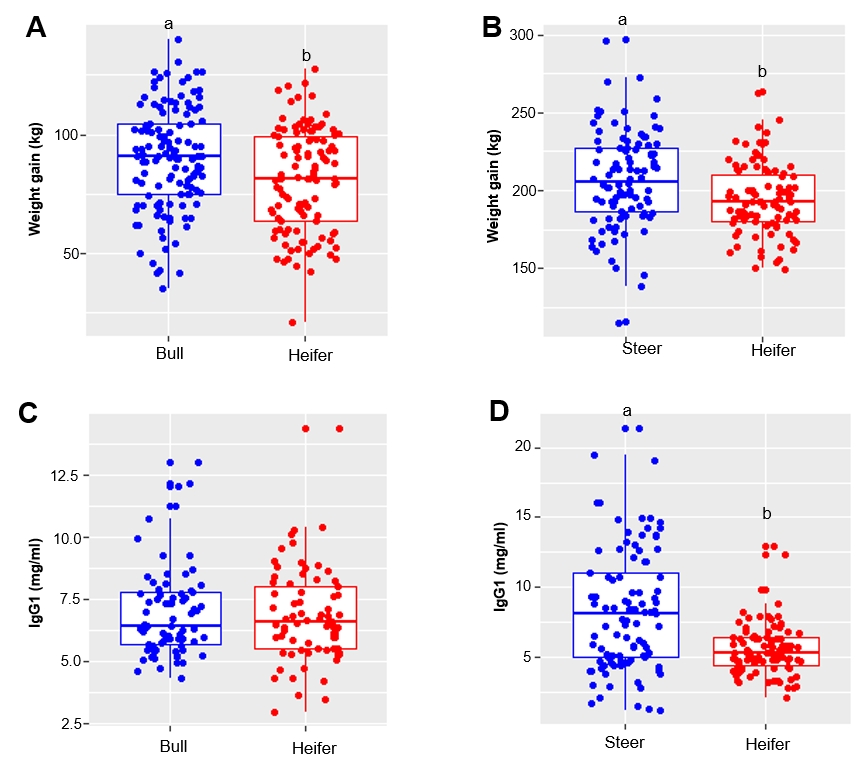


**Supplementary Figure 4. Differences in weight gain and IgG1 levels in blood plasma between heifers and bull (or steers) during preweaning and postweaning stages.** (A) Weight gain of preweaning heifers and bulls. (B) Weight gain of postweaning heifers and steers. (C) IgG1 levels in blood plasma of preweaning heifers and bulls. (D) IgG1 levels in blood plasma of postweaning heifers and steers. The statistical differences in weight gain and IgG1 between heifers and bulls (or steers) was analyzed using multiple linear regression models using breed composition, age, and sex as explanatory variables and weight gain and IgG1 levels in blood plasma as dependent variables. Different letters denote significant differences between groups.
